# Impact of sand and dust storms on mortality in Jinan City, China

**DOI:** 10.3389/fpubh.2025.1535543

**Published:** 2025-01-23

**Authors:** Chaofan Shen, Mingjun Li, Qingchang Wang, Jinjiao Luan, Jiliang Si, Liangliang Cui

**Affiliations:** ^1^School of Public Health, Cheeloo College of Medicine, Shandong University, Jinan, Shandong, China; ^2^Jinan Municipal Center for Disease Control and Prevention, Jinan, Shandong, China; ^3^Jinan Mental Health Center, Jinan, Shandong, China

**Keywords:** dust storms, PM_10_, mortality, case-crossover study, logistic regression

## Abstract

**Background:**

Sand and dust storms (SDSs) cause considerable health risks worldwide. China is a country seriously affected by SDSs, however only few studies researched the risk of SDS in China. The insufficient evidence on SDS hampers effective measures to mitigate its harm.

**Objective:**

To reveal the mortality risks associated with SDSs in Jinan City and identify sensitive populations vulnerable to these events.

**Methods:**

For this time-stratified case-crossover study, we collected daily data on all-cause, circulatory, and respiratory deaths, as well as air pollution and meteorological information from Jinan City in China between January 1, 2013, and November 30, 2022. We initially utilized a time-stratified case-crossover design and logistic regression model to examine the short-term relationship between SDSs and mortality risks, adjusting for specific variables such as mean temperature, humidity, wind speeds, and holidays. Subsequently, we conducted stratified analyses by age, gender, and season.

**Results:**

A total of 53 SDSs were observed, lasting for 88 days during the study period, which accounted for 2% of the study period. The excess mortality risks associated with SDSs were 13% (95% CI: 4–22%), 4% (95% CI: 1–8%), and 3% (95% CI: 1–6%) for respiratory, circulatory, and all-cause death, respectively. Females and people over 65 years of age are vulnerable to respiratory deaths caused by SDSs.

**Conclusion:**

Short-term exposure to SDSs caused the significantly elevated risks of respiratory, circulatory and all-cause death. Females and individuals over the age of 65 are particularly vulnerable to the effects of SDSs.

## 1 Introduction

Sand and dust storms (SDSs) are meteorological events caused by the ongoing release of significant amounts of mineral sand and dust particles into the atmosphere during specific favorable meteorological and synoptic conditions (1, 2). Generally, sand and dust particles were transported from one place to another by wind (3).

Poor air quality caused by SDSs threatens over 150 countries worldwide (4). The prevalence of SDSs has raised significant concern due to their harmful effects on human health (5, 6). Current investigations into the relationship between SDSs and health have primarily concentrated on the impact of SDS events on hospitalization and mortality rates. Research has shown that SDSs were notably linked to hospitalization rates in China (7, 8) and the Canary Islands, Africa (9). Independent studies from North America (10), Europe (11), and Oceania (12) indicated that SDSs increased non-accidental mortality. Several studies in East Asia have revealed that SDSs significantly raised all-cause and circulatory death rates (13–15). A recent study (16) demonstrated that exposure to SDS events was associated with an increased risk of circulatory and respiratory mortality in China, Asia.

Jinan City is located in the eastern part of China that is vulnerable to the effects of SDSs (16), with a population over 9 million. However, there is no study to investigate the effect of SDSs passing through Jinan City on mortality risks. To compensate for the limitation, this study explored the effects of SDSs passing through Jinan City on the risks of respiratory, circulatory, and all-cause death in the population based on a decade of mortality data in the city.

## 2 Materials and methods

### 2.1 Study area

This study area, Jinan City, is located in the mid-western of Shandong Province in Eastern China with low north high terrain south. It has a population of 9 million. The geographic position is between 36^°^01^′^N ~ 37^°^32^′^N and 116^°^11^′^E ~ 117^°^44^′^E. It belongs to typical warm temperate continental monsoonal climate zone that is characterized by a pronounced monsoon, four distinct seasons, a dry spring with little rain, a warm and rainy summer, a cool and dry autumn and a cold and little snow in winter. The perennial dominant wind direction of the city is from the southeast and east-southeast.

### 2.2 Data sources

We obtained death records from the China Cause of Deaths Reporting System (CDRS) and categorized causes using the International Classification of Diseases 10th Revision (ICD-10). Our dataset covered death from all-cause, circulatory diseases (ICD-10 codes I00-I99), and respiratory diseases (ICD-10 codes J00-J99).

The assessment of air pollution’s impact on mortality was conducted by analyzing the concentrations of various air pollutants: coarse particulate matter (PM_10_), fine particulate matter (PM_2_._5_), sulfur dioxide (SO_2_), nitrogen dioxide (NO_2_), carbon monoxide (CO), and 8-h ozone (O_3_-8h). There were 28 urban air quality monitoring stations to carry out real-time monitoring of these pollutants. They covered all the areas of Jinan City, whose sites are shown in Supplementary Table S1. Data of air pollutants were from the Jinan Ecological Environmental Protection Bureau website.^1^

Meteorological information, such as daily mean temperature (T, ^°^C), average relative humidity (RH, %), average air pressure (P, hPa), and average wind speeds (Wind, m/s), was collected from the China Meteorological Science Data Sharing Service Network.^2^ All data above were from the period between January 1, 2013, and November 30, 2022.

### 2.3 SDS definition

In this study, referring to the related study, SDS day was defined as day with a daily PM_10_ concentration exceeding 400 μg/m^3^ and a PM_2_._5_ to PM_10_ concentration ratio below 0.4 (16, 17).

### 2.4 Backward airflow trajectory analysis

We obtained the Global Data Assimilation System (GDAS) meteorological dataset from https://www.ready.noaa.gov/index.php and used MeteoInfoMap software (version 3.7.2; Chinese Academy of Meteorological Sciences; Beijing, China) to calculate 24-h backward airflow trajectories of SDSs. In China, there are three major sources of SDSs affecting population’s health, including the Taklamakan Desert and deserts of Inner Mongolia in China, and deserts of Mongolia, with the Taklamakan Desert affecting its nearby regions (18, 19), the deserts of Inner Mongolia in China and Mongolia contribute mainly to SDSs affecting China’s inland. To align with the airflow trajectories of SDSs impacting Jinan City, we first inputted the GDAS dataset for the days when these SDSs occurred using the MeteoInfoMap software. Next, we filled in the date, longitude, latitude, and sampling point height information in the respective data fields to calculate and fit the trajectories of the SDSs. This method yielded a strong simulation of the various source trajectories of SDSs. SDSs locations were identified based on their passage through Inner Mongolia in China, Mongolia or other areas, and their direction were recognized based on SDSs locations relative to Jinan City (Supplementary Table S2).

### 2.5 Statistical analyses

Firstly, we conducted descriptive analysis of the data, presenting indicators such as minimum (Min), maximum (Max), median (M), first quartile (P_25_), and third quartile (P_75_). Secondly, a time-stratified case-crossover study and logistic regression model was performed to evaluate the association between exposure to SDSs and mortality risks. The specific variables of mean temperature, humidity, wind speeds, and holidays were adjusted in the model. The design principle of a time-stratified case-crossover study is to stratify time, comparing the case phase and control phase within the same month, thus avoiding the confounding effects of long-term temporal trends. The control phase was selected to correspond to the same weekday of the other weeks within the same month and year as the case phase (e.g., if the SDS day occurred on the Wednesday of the 4th week of February 2013, the control days are chosen as the Wednesdays of the 1st, 2nd, and 3rd weeks of February 2013). The logistic regression model is a predictive tool used to estimate the probability of occurrence of the response variable, which varies with the dependent variables. We utilized the Wilcoxon rank-sum test to compare mortality risks between SDS days and non-SDS days.

Referring to the model in the related studies (16, 17), we determined the main model (Equation 1) in this study. It was as follows:


(1) $$\begin{array}{l} \log\left[E\left(Y_{t}\right)\right]=\alpha +\beta Z_{t}+\mathrm{ns}\left(Temp_{t},df\right)+\mathrm{ns}\left(RH_{t},df\right)+\\ \mathrm{ns}\left(\mathrm{Win}d_{t},df\right)+\mathrm{factor}\left(\mathrm{stratum}\right)+\\ \mathrm{factor}\left(\mathrm{holiday}\right) \end{array}$$

The definition of each variable in the model is shown in Supplementary material.

The following (Equation 2) calculated odds ratio (OR) for mortality associated with SDS events basing on the estimated *β* coefficients:


(2) $$\mathrm{OR}=e^{\left(\beta \right)}$$

### 2.6 Stratified analyses

Moreover, stratified analyses were conducted based on season (spring and winter), age (<65 and ≥ 65), and gender (males and females). Statistical differences between stratified estimates were estimated by two-sample Z-tests with the following formula (Equation 3):


(3) $$\left(\beta_{1}-\beta_{2}\right)/\sqrt{\left(S{E_{1}}^{2}+S{E_{2}}^{2}\right)}$$

β_1_ and β_2_ are regression coefficients specific to two subgroups. SE_1_ and SE_2_ are their corresponding standard errors.

### 2.7 Definition of lag days

We investigated the delayed impact of 31 days after the SDS, and observed that the risks of all-cause and circulatory death ceased by the 6th day after SDSs (lag 6), while the risk of respiratory death diminished at lag 3. Therefore, the lag days for both conditions were consistently identified as 6 days.

### 2.8 Sensitivity analyses

Sensitivity analyses were conducted by adjusting the degrees of freedom for the temperature variable (df = 7, 8, 9) and using different degrees of freedom for relative humidity and wind speed variables (df = 4, 5, 6) in spline functions (16). In addition, three alternative definitions of SDSs were tested by altering the PM_2_._5_ to PM_10_ concentration ratio (0.35 and 0.45) or considering only PM_10_ concentration (16).

Statistical analyses were performed using Rstudio software (version 4.2.3; Posit Inc., MA, United States). All tests were two-sided with statistical significance set at a *p*-value less than 0.05.

## 3 Results

### 3.1 Summary statistics for SDSs and mortality due to SDSs

**Figure 1:**
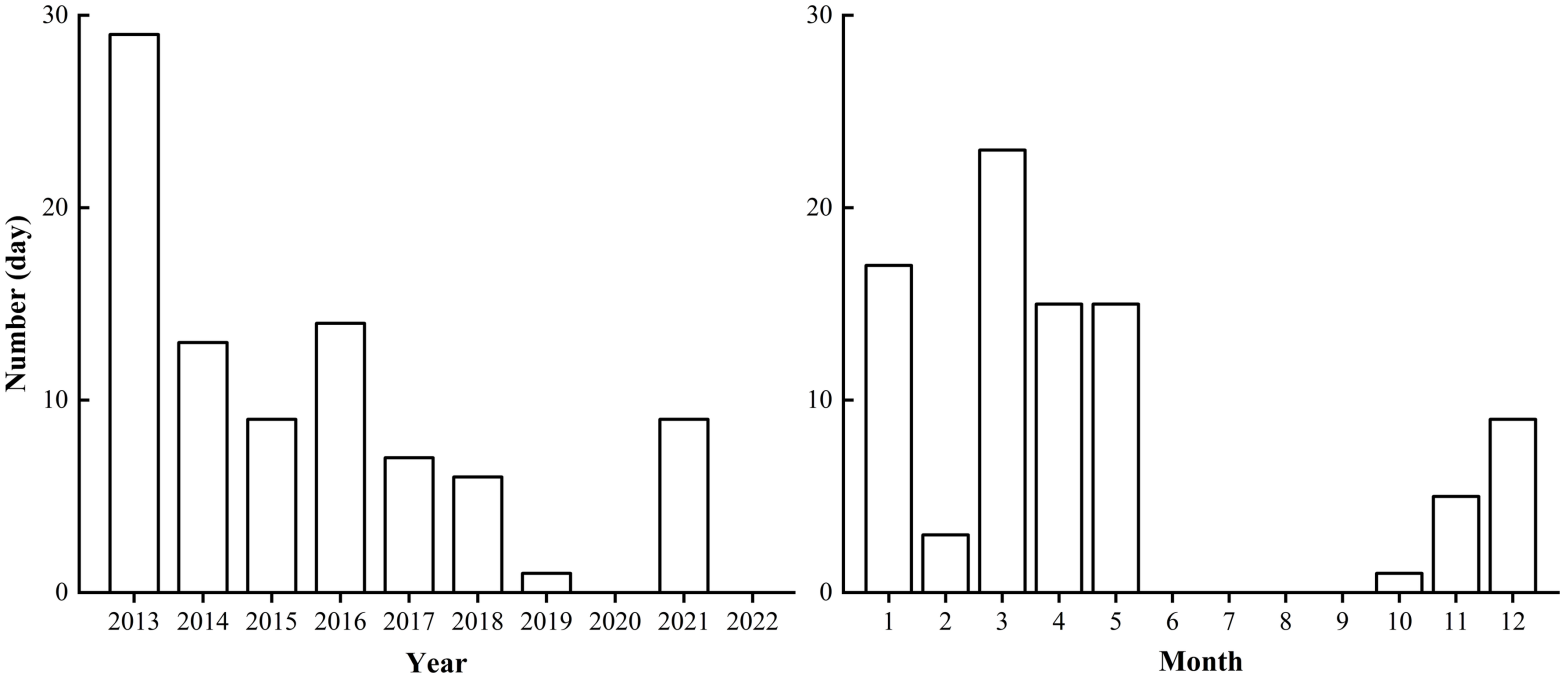
The yearly and monthly emergence number of sand and dust storms from 2013 to 2022 in Jinan City, China.

During the 10-year study period, 53 SDSs were recorded, with a duration of 88 days, representing 2% of the total study period. Figure 1 showed the annual emergence number of SDSs which primarily transpired from March to May and from November to January of the subsequent year.

**Figure 2:**
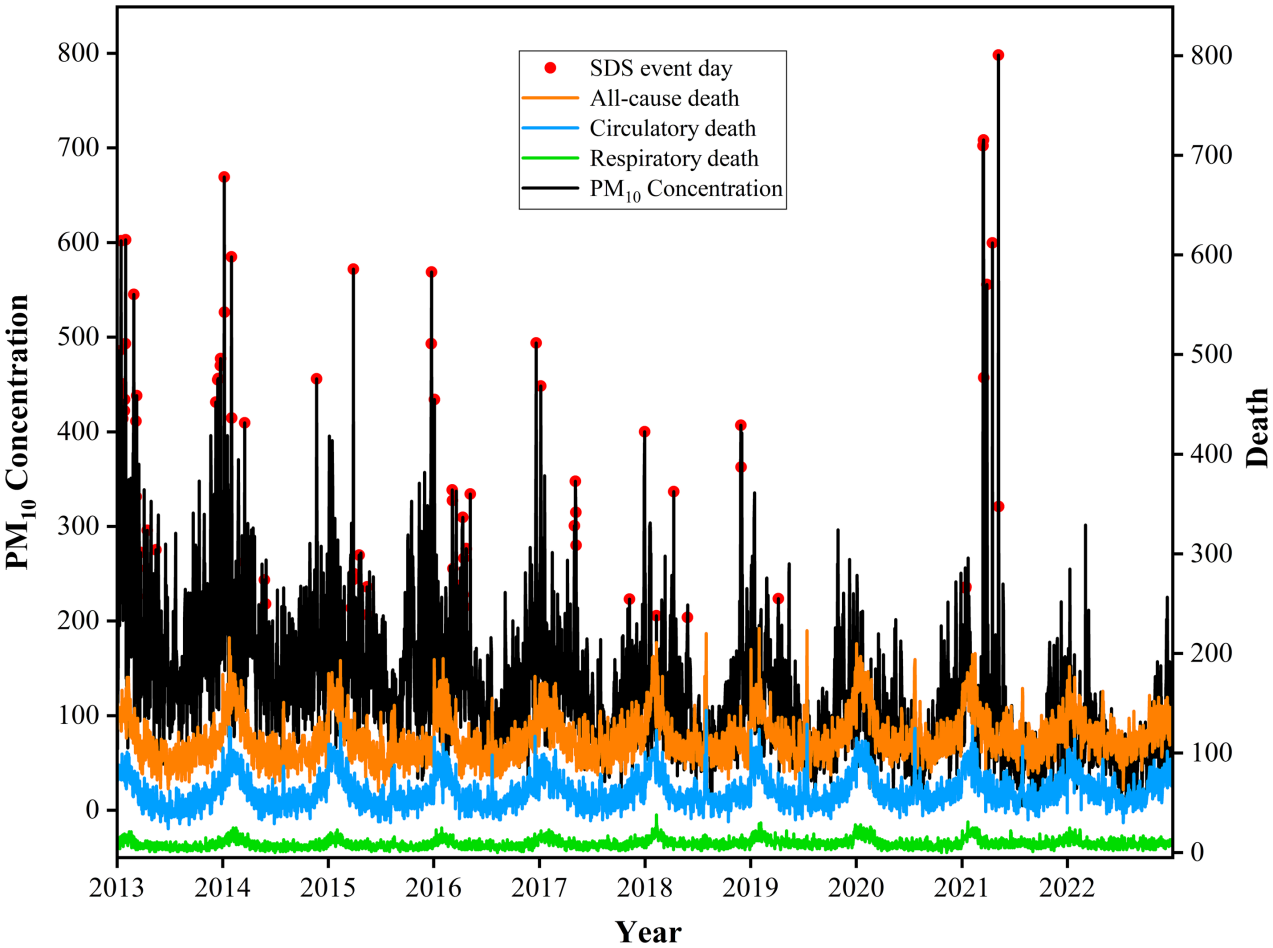
Temporal trends of death due to all-cause, circulatory, and respiratory with the concentration of PM_10_ during SDSs from 2013 to 2022 in Jinan City, China. Red Points represent the SDSs days; Black line represents PM_10_ concentration; Orange line represents all-cause death; Blue line represents circulatory death; Green line represents respiratory death. SDSs = sand and dust storms; PM_10_ = coarse particulate matter.

The demographic characteristics of deaths, meteorological factors, and air pollutants during SDS days and non-SDS days were displayed in Table 1. The number of deaths and the PM_10_ concentrations significantly increased during the study period (Figure 2). During SDSs, the concentrations of PM_10_, PM_2.5_, SO_2_, NO_2_, and CO were notably elevated compared to non-SDS days, whereas levels of O_3_ significantly decreased (Supplementary Table S3). Additionally, the daily death counts of all-cause, circulatory, and respiratory showed a significant elevation on SDSs days in comparison to non-SDS days (Supplementary Table S4).

**Table 1:** Summary statistics of mortality of all-cause, circulatory and respiratory, meteorological and air pollutants variables during SDSs and non-SDS days from 2013 to 2022 in Jinan city, China.

Variable	SDS days						Non-SDS days					
	n (%)	Min	P_25_	M	P_75_	Max	n (%)	Min	P_25_	M	P_75_	Max
All-cause death counts	10,572 (100)	76	104	116	132	211	407,090 (100)	62	99	111	126	225
<65 year	2,794 (26)	17	26	31	36	55	104,295 (26)	8	25	29	34	54
≥65 year	7,778 (74)	44	75	86	97	178	302,795 (74)	37	72	82	96	178
Male	5,817 (55)	36	57	66	73	103	227,293 (56)	31	55	63	71	127
Female	4,755 (45)	33	45	52	60	108	179,797 (44)	24	42	49	58	119
Circulatory death counts	5,846 (100)	33	57	64	73	123	216,915 (100)	24	50	59	70	143
<65 year	1,069 (18)	3	9	12	14	23	37,649 (17)	2	8	10	13	26
≥65 year	4,777 (82)	23	48	52	61	113	179,266 (83)	17	41	48	58	118
Male	2,940 (50)	13	28	32	39	56	111,130 (51)	8	25	30	37	79
Female	2,906 (50)	16	28	32	38	68	105,785 (49)	9	23	29	35	73
Respiratory death counts	907 (100)	2	7	10	13	38	32,882 (100)	0	6	8	12	31
<65 year	88 (10)	0	0	1	2	4	3,246 (10)	0	0	1	1	7
≥65 year	819 (90)	1	6	9	12	34	29,576 (90)	0	5	8	11	28
Male	485 (53)	1	3	5	7	18	17,690 (54)	0	3	5	7	19
Female	422 (47)	0	3	5	6	20	15,192 (46)	0	2	4	6	17
Meteorological												
RH (%)	88 (−)	18	33	47	65	97	3,533 (−)	15	41	55	70	100
Mean.T. (°C)	88 (−)	-3	3	13	19	33	3,533 (−)	-12	6	17	25	34
Pressure (hPa)	88 (−)	981	992	997	1,003	1,013	3,533 (−)	975	988	997	1,004	1,022
Wind (m/s)	88 (−)	1	2	2	3	8	3,533 (−)	0	2	2	3	8
Air pollution												
PM_10_ (μg/m^3^)	88 (−)	199	244	332	456	798	3,533 (−)	5	75	111	158	399
PM_2.5_ (μg/m^3^)	88 (−)	76	88	104	264	443	3,533 (−)	4	33	51	83	280
SO_2_ (μg/m^3^)	88 (−)	7	37	66	149	429	3,533 (−)	5	12	21	42	382
CO (μg/m^3^)	88 (−)	391	1,033	1,426	3,381	6,555	3,533 (−)	277	707	925	1,232	5,102
NO_2_ (μg/m^3^)	88 (−)	15	46	59	92	165	3,533 (−)	9	29	40	54	137
O_3_ (μg/m^3^)	88 (−)	11	27	84	112	238	3,533 (−)	7	62	100	149	282
PM_2.5_/PM_10_	88 (−)	0.2	0.4	0.4	0.6	0.8	3,533 (−)	0.1	0.4	0.5	0.6	0.9

SDSs, sand and dust storms; Min, Minimum; P_25_, 25th percentile; M, Median; P_75_, 75th percentile; Max, Maximum; Mean.T., Mean Temperature; RH, Relative humidity; PM_2.5_, Fine particulate matter; PM_10_, Coarse particulate matter; SO_2_, Sulfur dioxide; CO-Carbon monoxide; NO_2_, Nitrogen dioxide; O_3_, Ozone.

After calculating the backward airflow trajectories of SDSs passing through Jinan City, the study classified the source locations of SDSs into Mongolia (9, 17%); Inner Mongolia in China (18, 34%); Inner Mongolia in China and Mongolia (16, 30%); and other regions (10, 19%). The transportation routes identified were northwest (41, 77%); northeast (8, 15%); southwest (2, 4%); and west (2, 4%) (Supplementary Figure S1).

### 3.2 Association between SDSs and mortality due to SDSs

**Figure 3:**
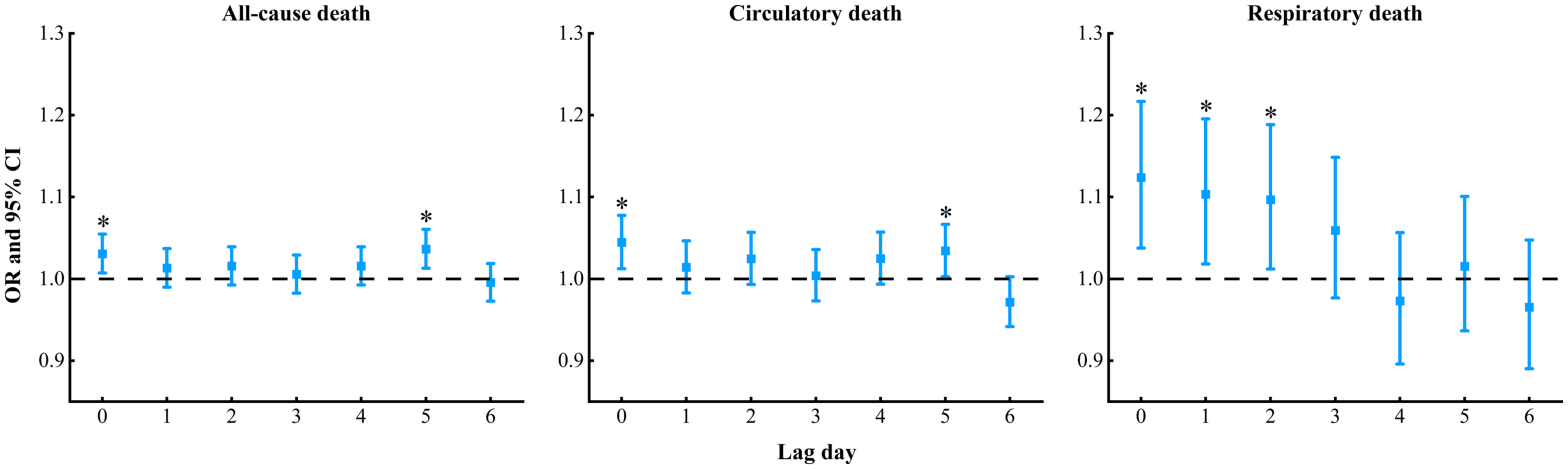
Summary of lag effect of sand and dust storms on the risks of all-cause, circulatory and respiratory death from 2013 to 2022 in Jinan City, China. The “*” represents statistically significant.

A significant increase in the risks of respiratory, circulatory and all-cause death are shown in Figure 3, with the highest death risk observed at lag0 [odds ratio (OR) = 1.13, 95% confidence interval (CI): 1.04, 1.22], lag0 (OR = 1.04, 95% CI: 1.01, 1.08), lag5 (OR = 1.03, 95% CI: 1.01, 1.06), respectively.

### 3.3 Stratified analyses results

**Figure 4:**
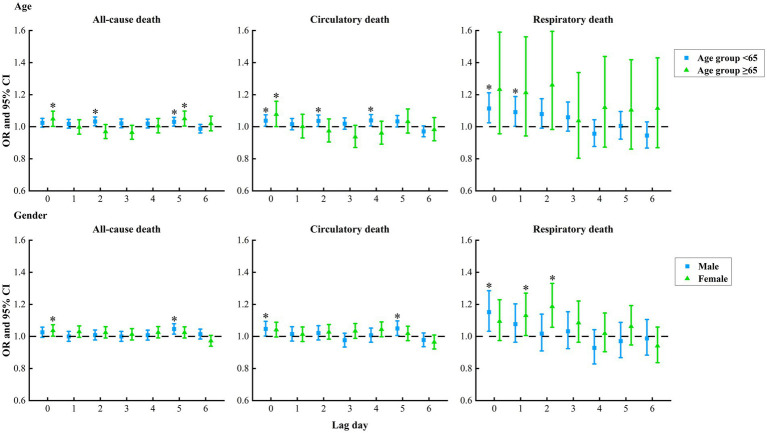
Subgroups analysis of lag effect of sand and dust storms on the risks of all-cause, circulatory and respiratory death from 2013 to 2022 in Jinan City, China. The “*” represents statistically significant.

In subgroups analysis of age, we observed that the risk of respiratory death associated with SDSs in the age group ≥65 was higher than that in the age group <65, with the maximum lag effect in the age group ≥65 emerged on lag2 (OR = 1.25, 95% CI: 0.98, 1.60), and that in the age group <65 occurred on lag0 (OR = 1.12, 95% CI: 1.03, 1.22). The risks of all-cause death were notably increased in both age groups, with the maximum lag effect in the age group ≥65 appeared on lag2 (OR = 1.05, 95% CI: 1.01, 1.10), and that in the age group <65 occurred on lag5 (OR = 1.03, 95% CI: 1.01, 1.06), but their group differences were not significant. Meanwhile, the risks of circulatory death were significantly elevated in both age groups, with the maximum lag effect in the age group ≥65 occurred on lag4 (OR = 1.07, 95% CI: 1.01, 1.15), and that in the age group <65 emerged on lag0 (OR = 1.04, 95% CI: 1.01, 1.08), but their group differences were not significant (Figure 4).

Additionally, it was observed that the risk of respiratory death was higher in females compared to males, with the highest risk in females occurring at lag2 (OR = 1.19, 95% CI: 1.06, 1.34) and in males at lag0 (OR = 1.16, 95% CI: 1.04, 1.29). There was a significant increase in the risks of all-cause death in both genders, with the highest risk in males at lag5 (OR = 1.05, 95% CI: 1.02, 1.08) and in females at lag0 (OR = 1.04, 95% CI: 1.01, 1.07), although the differences between the groups were not significant. Additionally, the risks of circulatory death significantly rose in both genders, with the highest risk in males at lag5 (OR = 1.05, 95% CI: 1.01, 1.10) and in females at lag4 (OR = 1.04, 95% CI: 1.00, 1.09), but the group differences were not significant (Figure 4).

**Figure 5:**
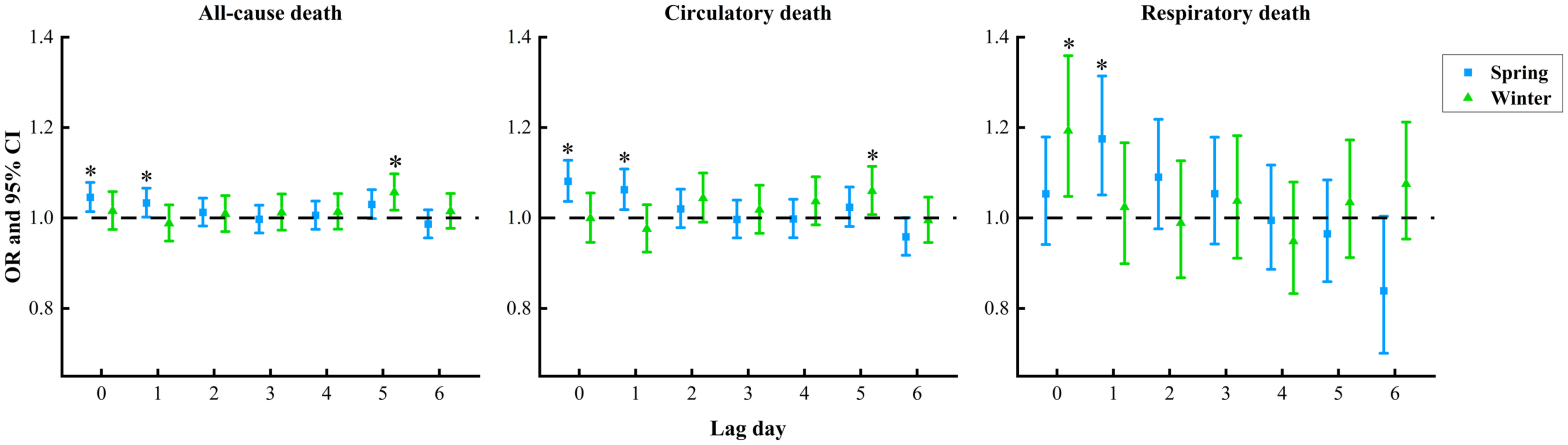
Summary of lag effect of sand and dust storms during the different seasons on the risks of all-cause, circulatory and respiratory death from 2013 to 2022 in Jinan City, China. The “*” represents statistically significant.

In a stratified analysis of the seasons, we observed that SDSs in the two seasons notably increased risks of all-cause, circulatory, respiratory death, but the differences of these groups were not significant. The maximum lag effect of all-cause death in the spring appeared on lag0 (OR = 1.05, 95% CI: 1.01, 1.08), while that in the winter occurred on lag5 (OR = 1.06, 95% CI: 1.02, 1.10). The maximum lag effect of circulatory death in the spring appeared on lag0 (OR = 1.08, 95% CI: 1.04, 1.13), while that in the winter occurred on lag5 (OR = 1.06, 95% CI: 1.01, 1.11). The maximum lag effect of respiratory death in the spring appeared on lag1 (OR = 1.18, 95% CI: 1.05, 1.31), while that in the winter occurred on lag0 (OR = 1.19, 95% CI: 1.05, 1.36) (Figure 5).

### 3.4 Sensitive analyses results

The sensitivity analyses showed that the main findings remained nearly unchanged, suggesting that the main model had a good fit and produced stable results (Supplementary Figures S2, S3).

## 4 Discussion

We conducted a retrospective analysis to explore the association between SDSs passing through Jinan City and mortality risks over the past decade. We observed that SDSs passing through Jinan City originate from Inner Mongolia in China, Mongolia, or other regions. Meanwhile, Jinan City is a region prone to the impact of SDSs (16). Our findings indicated a notable rise in the risks of respiratory, circulatory, and all-cause death linked with SDSs. This is consistent with the study by Pouri et al. who observed that SDSs resulted in a 18%, 25%, and 16% elevated risk of respiratory, circulatory, and all-cause death, respectively (20). A previous study in China also demonstrated that SDSs lead to an elevated excess mortality risk from circulatory and respiratory diseases. They found an 8.9% elevated excess mortality risk for respiratory death due to SDSs (16), which was lower than the result of our study in Jinan City, suggesting that SDSs passing through Jinan City were even more dangerous and needed attention.

In line with a previous study (20), our study revealed that the older adult are more vulnerable to respiratory death due to SDSs. The increased vulnerability of the older adult to air pollution can be attributed to the natural deterioration of the immune system with age (21). This decline in immune function reduces their ability to resist environmental hazards effectively (22–24). In addition, older people are more prone to chronic diseases, which can worsen their current diseases and even cause mortality (25). Older adult individuals with chronic obstructive pulmonary disease (COPD) faced increased mortality rates after exposure to outdoor air pollution (26, 27).

Our findings suggest that females face a heightened risk of respiratory death related to SDS events. The study of Pouri et al. (20) also revealed that SDSs notably elevated respiratory mortality in females. Several studies have proved that air pollution is more likely to have severe influences on females (28–31). These may be explained by gender variances in physiological structures that females have narrower airway dimensions and higher breathing rates (32). One study showed that females have a faster respiratory rate than males (33), which could be a possible reason why women are more susceptible to the effects of air pollution than men.

China is a country significantly affected by SDSs. With increasing awareness of the dangers posed by SDSs, various strategies have been proposed to mitigate the health risks associated with air pollution events, including SDSs (34–37).

## 5 Conclusion

Short-term exposure to SDSs caused the significantly elevated risks of respiratory, circulatory and all-cause death. Females and people over 65 years of age are vulnerable to respiratory deaths caused by SDSs. This study, conducted in Jinan City, offers new evidence regarding the adverse effects of SDSs on the risks of respiratory, circulatory, and all-cause mortality through a time-stratified case-crossover analysis.

## Data Availability

The original contributions presented in the study are included in the article/Supplementary material, further inquiries can be directed to the corresponding authors.

## Footnotes

^1^ http://fb.sdem.org.cn:8801/airdeploy.web/AirQuality/MapMain.aspx

^2^ http://data.cma.cn/
