# Supplementary material for: Impact of sand and dust storms on mortality in Jinan City, China
Source: Front Public Health. 2025 Jan 23;13:1535543. doi: 10.3389/fpubh.2025.1535543 (PMC11799952; doi:10.3389/fpubh.2025.1535543)
Supplement: Supplementary file 1 [file Data_Sheet_1.docx]

***Supplementary Material***

1. **logistic regressions model**

$$\log\left[ E\left( Y_{t} \right) \right] =\alpha+\beta Z_{t}+ns\left( {Temp}_{t}, df \right)+ns\left( {RH}_{t},df \right)+ns\left( {Wind}_{t},df \right)+factor\left( stratum \right)+factor\left( holiday \right) \left( 1 \right)$$

In the study, each variable is defined as follows: t represents the day of observation, Y_t_ represents the number of deaths on day t, Z_t_ represents the level of exposure on day t, with “1” indicating an identified SDS day and “0” for a non-SDS day. The parameters include α as the intercept term, β as the coefficient vector. The natural spline functions (ns) were applied to control confounding factors like daily mean temperature (Temp_t_), daily average relative humidity (RH_t_), and daily average wind speeds (Wind_t_) with degrees of freedom (df) set at 6, 3, and 3, respectively, determined by Akaike informativeness criterion (AIC) (1, 2) and prior research (3, 4). Stratum is a categorical variable matching year, month, and week; holiday variables were also considered.

**References**

1. Hurvich CM, Simonoff JS, Tsai CL. Smoothing parameter selection in nonparametric regression using an improved Akaike information criterion. *Journal of the Royal Statistical Society Series B-Statistical Methodology*. (1998) 60:271-293. doi: 10.1111/1467-9868.00125

2. Shi PD, Tsai CL. A note on the unification of the Akaike information criterion. *Journal of the Royal Statistical Society Series B-Statistical Methodology*. (1998) 60:551-558. doi: 10.1111/1467-9868.00139

3. Jung J, Lee EM, Myung W, Kim H, Kim H, Lee H. Burden of dust storms on years of life lost in Seoul, South Korea: A distributed lag analysis. *Environ Pollut*. (2022) 296:118710. doi: 10.1016/j.envpol.2021.118710

4. Zhang C, Yan ML, Du H, Ban J, Chen C, Liu YY, et al. Mortality risks from a spectrum of causes associated with sand and dust storms in China. *Nat commun*. (2023) 14(1):6867. doi: 10.1038/s41467-023-42530-w

1. **Supplementary Tables**

| **Supplementary Table 1** The names and their affiliation areas of air quality monitoring stations in Jinan City | | |
| --- | --- | --- |
| **Number** | **Site name** | **affiliated region** |
| 1 | Jinan Chemical Factory | Tianqiao district |
| 2 | Lixia District Development Zone | Lixia district |
| 3 | Jinan Science and Technology Commission Retired Cadres Recreation Center | Shizhong district |
| 4 | Jinan Institute of Agricultural Science | Huaiyin district |
| 5 | Shandong Province Seed Warehouse | Licheng district |
| 6 | Jinan Monitoring Station | Lixia district |
| 7 | Changqing District Party School | Changqing district |
| 8 | Shandong Construction Engineering College | Licheng district |
| 9 | Shandong Economic College | Lixia district |
| 10 | Lanxiang Technical School | Tianqiao district |
| 11 | Quan Cheng Plaza | Lixia district |
| 12 | Shandong Luneng Group | Shizhong district |
| 13 | The Second Machine Tool Factory | Shizhong district |
| 14 | Jinan western urban area | Huaiyin district |
| 15 | Jiyangcheng urban area | Jiyang district |
| 16 | Jiyang Development Zone | Jiyang district |
| 17 | Jinping Middle School | Lixia district |
| 18 | Pingyin urban area | Pingyin county |
| 19 | Pingyin Development Zone | Pingyin county |
| 20 | Shanghe urban area | Shanghe county |
| 21 | Shanghe Development Zone | Shanghe county |
| 22 | Shandong Institute of Commerce and Technology | Licheng district |
| 23 | Licheng District Experimental School | Licheng district |
| 24 | Jinan Museum | Lixia district |
| 25 | Zhangqiu urban area | Zhangqiu district |
| 26 | Zhangqiu Development Zone | Zhangqiu district |
| 27 | Changqing College Town | Changqing district |
| 28 | Paoma Mountain | Licheng district |

| **Supplementary Table 2** The summary of original locations and transport routes of sand and dust storms from 2013 to 2022 | | |
| --- | --- | --- |
| **Date** | **Original location** | **Transportation route** |
| 2013-01-08 | Inner Mongolia in China | Northwest |
| 2013-01-13 | Inner Mongolia in China | Northwest |
| 2013-01-20 | Inner Mongolia in China | Northeast |
| 2013-01-26 | Inner Mongolia in China | Northwest |
| 2013-02-27 | Other areas | Northwest |
| 2013-03-03 | Inner Mongolia in China and Mongolia | Northwest |
| 2013-03-07 | Other areas | Northwest |
| 2013-03-23 | Mongolia | Northwest |
| 2013-04-14 | Mongolia | Northwest |
| 2013-05-01 | Other areas | Northwest |
| 2013-05-15 | Inner Mongolia in China | Northeast |
| 2013-11-16 | Inner Mongolia in China and Mongolia | Northwest |
| 2013-12-08 | Mongolia | Northwest |
| 2013-12-15 | Inner Mongolia in China | Northwest |
| 2013-12-24 | Inner Mongolia in China | Northwest |
| 2014-01-01 | Inner Mongolia in China and Mongolia | Northwest |
| 2014-01-06 | Other areas | Northeast |
| 2014-01-31 | Inner Mongolia in China | Northwest |
| 2014-03-17 | Inner Mongolia in China and Mongolia | Northwest |
| 2014-03-31 | Inner Mongolia in China | Northeast |
| 2014-05-25 | Inner Mongolia in China and Mongolia | Northwest |
| 2014-05-29 | Mongolia | Northwest |
| 2014-10-15 | Inner Mongolia in China and Mongolia | Northwest |
| 2014-11-22 | Other areas | Northwest |
| 2015-03-21 | Inner Mongolia in China and Mongolia | Northwest |
| 2015-03-28 | Mongolia | Northwest |
| 2015-04-18 | Inner Mongolia in China | Northeast |
| 2015-05-15 | Inner Mongolia in China | Northeast |
| 2015-12-23 | Inner Mongolia in China | Northwest |
| 2016-01-03 | Inner Mongolia in China | Northwest |
| 2016-03-05 | Inner Mongolia in China | Northwest |
| 2016-03-22 | Inner Mongolia in China | Northeast |
| 2016-03-31 | Inner Mongolia in China and Mongolia | Northwest |
| 2016-04-10 | Inner Mongolia in China | Northwest |
| 2016-04-22 | Inner Mongolia in China and Mongolia | Northwest |
| 2016-05-06 | Inner Mongolia in China and Mongolia | Northwest |
| 2016-12-20 | Inner Mongolia in China | Northwest |
| 2017-01-05 | Other areas | Southwest |
| 2017-05-01 | Other areas | Northwest |
| 2017-05-05 | Inner Mongolia in China and Mongolia | Northwest |
| 2017-11-08 | Mongolia | Northwest |
| 2017-12-30 | Inner Mongolia in China and Mongolia | Northwest |
| 2018-02-09 | Inner Mongolia in China and Mongolia | Northwest |
| 2018-04-02 | Inner Mongolia in China | Northeast |
| 2018-04-10 | Mongolia | Northwest |
| 2018-05-27 | Other areas | West |
| 2018-11-28 | Other areas | Southwest |
| 2019-04-07 | Other areas | West |
| 2021-01-14 | Inner Mongolia in China and Mongolia | Northwest |
| 2021-03-15 | Mongolia | Northwest |
| 2021-03-28 | Inner Mongolia in China and Mongolia | Northwest |
| 2021-04-16 | Mongolia | Northwest |
| 2021-05-07 | Inner Mongolia in China and Mongolia | Northwest |

| **Supplementary Table 3** The daily average concentration of pollutants during SDSs days and non-SDSs days from 2013 to 2022 in Jinan city, China [M (P_25_-P_75_)] | | |
| --- | --- | --- |
| **Pollutants (μg/m^3^)** | **SDS day** | |
|  | **Yes (n=88 days)** | **No (n=3,533 days)** |
| PM_10_ | 332 (244-456)^*^ | 111 (75-158) |
| PM_2.5_ | 104 (88-264)^*^ | 51 (33-83) |
| SO_2_ | 66 (37-149)^*^ | 21 (12-42) |
| CO | 1,426 (1,033-3,381)^*^ | 925 (707-1,232) |
| NO_2_ | 59 (46-92)^*^ | 40 (29-54) |
| O_3_ | 84 (27-112)^*^ | 100 (62-149) |
| SDSs=sand and dust storms; M=Median; P_25_=25th percentile; P_75_=75th percentile; PM_2.5_=Fine particulate matter; PM_10_=Coarse particulate matter; SO_2_=Sulfur dioxide; CO=Carbon monoxide; NO_2_=Nitrogen dioxide; O_3_=Ozone. The “^*^” represents a statistically significant | | |

| **Supplementary Table 4** The daily average counts of all-cause, circulatory and respiratory death during SDSs from 2013 to 2022 in Jinan city, China [M (P_25_-P_75_)] | | |
| --- | --- | --- |
| **Death counts** | **SDSs day** | |
|  | **Yes (n=88 days)** | **No (n=3,533 days)** |
| All-cause death | 116 (104-132)^*^ | 111 (99-126) |
| <65 year | 31 (26-36)^*^ | 29 (25-34) |
| ≥65 year | 86 (75-97) | 82 (72-96) |
| Male | 66 (57-73) | 63 (55-71) |
| Female | 52 (45-60)^*^ | 49 (42-58) |
| Circulatory death | 64 (57-73)^*^ | 59 (50-70) |
| <65 year | 12 (9-14)^*^ | 10 (8-13) |
| ≥65 year | 52 (48-61)^*^ | 48 (41-58) |
| Male | 32 (28-39)^*^ | 30 (25-37) |
| Female | 32 (28-38)^*^ | 29 (23-35) |
| Respiratory death | 10 (7-13)^*^ | 8 (6-12) |
| <65 year | 1 (0-2) | 1 (0-1) |
| ≥65 year | 9 (6-12)^*^ | 8 (5-11) |
| Male | 5 (3-7) | 5 (3-7) |
| Female | 5 (3-6)^*^ | 4 (2-6) |
| SDSs=sand and dust storms; M=Median; P_25_=25th percentile; P_75_=75th percentile. The “^*^” represents a statistically significant | | |

1. **Supplementary Figures**

| 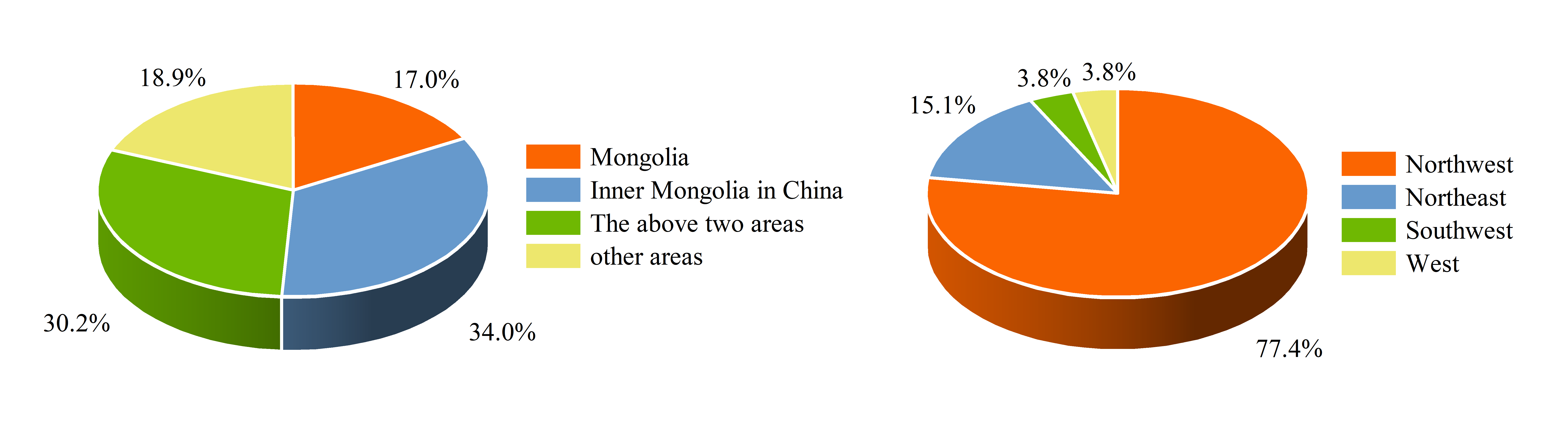 |
| --- |
| **Supplementary Figure 1** The summary description of the number of SDSs from different places of origin and different directions |

|  |
| --- |
| **Supplementary Figure 2** Sensitivity analyses for changing the definition of SDSs. SDSs=sand and dust storms; OR=Odds ratio; CI=Confidence interval. The “*” represents statistically significant |

| 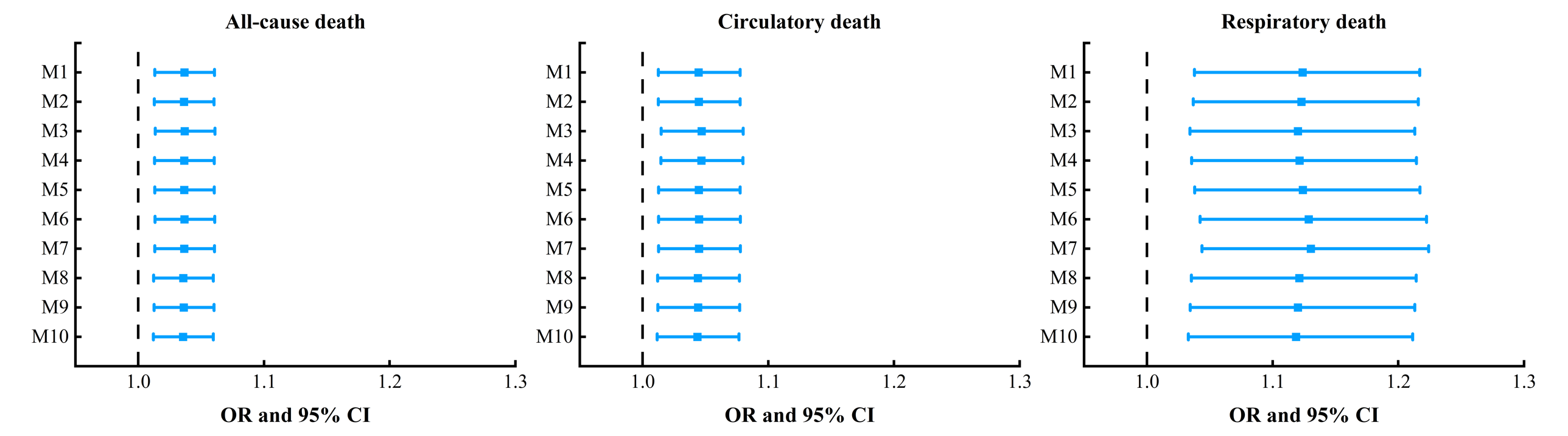 |
| --- |
| **Supplementary Figure 3** Sensitivity analyses for changing the degrees of freedom of variables. M1 represents main model; M2, M3, M4 represent the df = 7, 8, 9 for temperature in the models, respectively; M5, M6, M7 represent the df = 4, 5, 6 for relative humidity in the models, respectively; M8, M9, M10 represent the df = 4, 5, 6 for wind speeds in the models, respectively. OR=Odds ratio; CI=Confidence interval |
